# Flux-growth method for the targeted synthesis of the salt-inclusion copper(II) phosphate Rb_9_Na_2_Cu_6_(P_2_O_7_)_4_Cl_7_

**DOI:** 10.1107/S2056989026004287

**Published:** 2026-05-07

**Authors:** Karla Luviano, Emily D. Williams, Duminda S. Liurukara, Yasmin Dayeh, Julia Cox, Kulugammana G. S. Ranmohotti

**Affiliations:** a Division of Science Mathematics and Technology Governors State University, 1 University Parkway University Park IL 60484-0975 USA; b Department of Chemistry and Center for Optical Materials Science and Engineering Technologies (COMSET), Clemson University, Clemson, SC 29634-0973, USA; c University of Missouri Research Reactor (MURR) University of Missouri, Columbia, MO 65211 USA; Vienna University of Technology, Austria

**Keywords:** crystal structure, salt-inclusion solids, eutectic flux, molten-salt approach, open framework, salt structure, bond-valence-sum calculations

## Abstract

There is a great deal of inter­est in the synthesis and structural features of a newly emerged class of salt-inclusion solids (SISs), which are made of a hybrid framework exhibiting integrated covalent (metal oxides) and ionic (metal halides) structural units. In general, we explored the conditions under which desired phase formation occurs and, for this study, we selected one of the com­pounds in the series of salt-templated phosphates and arsenates, *A*_2_*M*_3_(*X*_2_O_7_)_2_·(salt) [where *A* = K, Rb, Cs; *M* = Mn, Cu; *X* = P, As] commonly known as CU-2 materials, which crystallize in two different space groups (*I*4/*mcm* and *P*4/*nbm*) due to the variation of the structural units. In our attempt to incorporate mixed Rb^+^/Na^+^ cations along with Cl^−^ anions into the negatively charged framework, Cu_3_(P_2_O_7_)_2_^2−^, the title com­pound, Rb_9_Na_2_Cu_6_(P_2_O_7_)_4_Cl_7_, nona­rubidium disodium hexa­copper(II) tetra­kis­(pyrophosphate) hepta­chloride, has been synthesized by employing a high-tem­per­a­ture RbCl/NaCl eutectic flux and structurally characterized by single-crystal X-ray diffraction, revealing *I*4/*mcm* as the space group. In the title com­pound, the same negatively charged framework, Cu_3_(P_2_O_7_)_2_^2–^, characteristic of the CU-2 materials, is retained, while the extended salt-like part of the structure, com­posed of mixed alkali metal chloride fragments, is different from the known CU-2 materials, and includes rarely seen corner-sharing {Na_4_Cl_8_} units.

## Abstract 2

The title com­pound, Rb_9_Na_2_Cu_6_(P_2_O_7_)_4_Cl_7_, is another member of the series of salt-templated phosphates and arsenates, *A*_2_*M*_3_(*X*_2_O_7_)_2_·(salt) [where *A* = K, Rb, Cs; *M* = Mn, Cu; *X* = P, As] commonly known as CU-2 materials. The structure exhibits rarely seen corner-sharing Na_4_Cl_8_ units.

## 1. Chemical context

Exploratory synthesis has been a vital aspect of solid-state chemistry, where the discovery of new com­pounds and structure types leads to new systems that can be optimized for a desired property and even to the discovery of new unexpected properties. These unprecedented discoveries have opened doors to novel materials synthesis *via* the utilities of salt-inclusion chemistry (SIC) that are otherwise known as the molten-salt approach (West & Hwu, 2012 ▸). The solubility of metal oxides in molten salts facilitates the synthesis of com­plex oxide com­pounds, such as transition-metal phosphates, arsenates, and silicates (Hwu, 1998 ▸). Although occasional salt inclusion is inevitable, this approach provides added variety in structural features of the resulting solids. These solids display an integrated structure between chemically dissimilar structures of a more covalent metal oxide and a more ionic halide salt.

By a broad definition, this class of materials is called salt-inclusion solids (SISs). Alternatively, the SISs are viewed as a metal oxide covalent framework templated by an extended structural unit made of an ionic salt (Hwu *et al.*, 2002 ▸). The characteristic feature of SISs is that the more covalent metal oxide framework consists of voids filled by ionic salt-like structural units exhibiting 0-periodicity (Tang *et al.*, 2008 ▸), 1-periodicity (Yu *et al.*, 2013 ▸), or 2-periodicity (Queen *et al.*, 2008 ▸). These salt-like units usually have com­plimentary structural features with respect to the negatively charged metal oxide framework (Hwu & Mo, 2001 ▸). It is intriguing to notice that the shape of the pore windows can be varied by altering the identity and relative concentration of the incorporated salt (Huang & Hwu, 2003 ▸). Recent reports of SISs have highlighted a correlation between the incorporation of ionic salts and the formation of special frameworks that would otherwise not be isolated without the aid of molten salt as a reactive solvent in the synthesis of metal oxide frameworks with low periodicity (Morrison *et al.*, 2016*a* ▸). Many of the SISs adopt new structure types, whereby the incorporated salts play an essential role in bulk structural and chemical/physical properties. Most of the fascinating physical properties exhibited in SISs are associated with features like porous frameworks (Ulutagay *et al.*, 1998 ▸), non-centrosymmetric structures (Etheredge & Hwu, 1995 ▸), or magnetic nanostructures (Stern *et al.*, 2006 ▸). Inter­estingly, in some cases, SISs exhibit intense luminescence (Morrison *et al.*, 2016*b* ▸) and can have important applications as new waste forms for the safe long-term storage of radio isotopes (Morrison & zur Loye, 2016 ▸).

With increasing inter­est, the above findings convey the fact that salt inclusion is a valid tool for a broad range of synthetic chemistry, and the SISs represent a newly emerging class of solids. However, SISs have remained a challenge to synthesize as their synthesis is largely serendipitous (Gao *et al.*, 2015 ▸). Therefore, it is important to shift the focus of this exploration toward the development of new synthesis routes that allow for the targeted growth of new com­pounds within already explored phase and com­positional space. Inspired by the study of salt-templated phosphates and arsenates of the type *A*_2_*M*_3_(*X*_2_O_7_)_2_·(salt) (where *A* is K, Rb, Cs; *M* is Mn, Cu; *X* is P, As), commonly known as CU-2 materials (Huang *et al.*, 1999 ▸), K_1.23_Cs_3.60_Mn_3_(P_2_O_7_)_2_Cl_3.74_, K_2.12_Cs_2.76_Mn_0.76_Cu_2.24_(P_2_O_7_)_2_Cl_2.87_, K_3.81_Cs_1.44_Cu_3_(P_2_O_7_)_2_Cl_3.25_, and Rb_1.14_Cs_4.15_Cu_3_(As_2_O_7_)_2_Cl_3.19_, designated as CU-2-MnPO, CU-2-MnCuPO, CU-2-CuPO, and CU-2-CuAsO, respectively, we have undertaken an investigation of the salt-inclusion type CU-2-CuPO phase. The aim of this work is to design a reaction taking place by the careful selection of a metal oxide mixture aiming at the Rb_2_Cu_3_(P_2_O_7_)_2_ com­position and the use of a mixed RbCl/NaCl eutectic flux to isolate salt-inclusion com­pounds of the form Rb_2–*x*_Na_*x*_Cu_3_(P_2_O_7_)_2_·*y*(RbCl). Here we report a second member of CU-2-CuPO materials of which the idealized formula can be written as Rb_2_Na_2_Cu_6_(P_2_O_7_)_4_·7(RbCl), where the open framework is conceptually templated by extended (Rb/Na)Cl units.

## 2. Structural commentary

**Figure 1:**
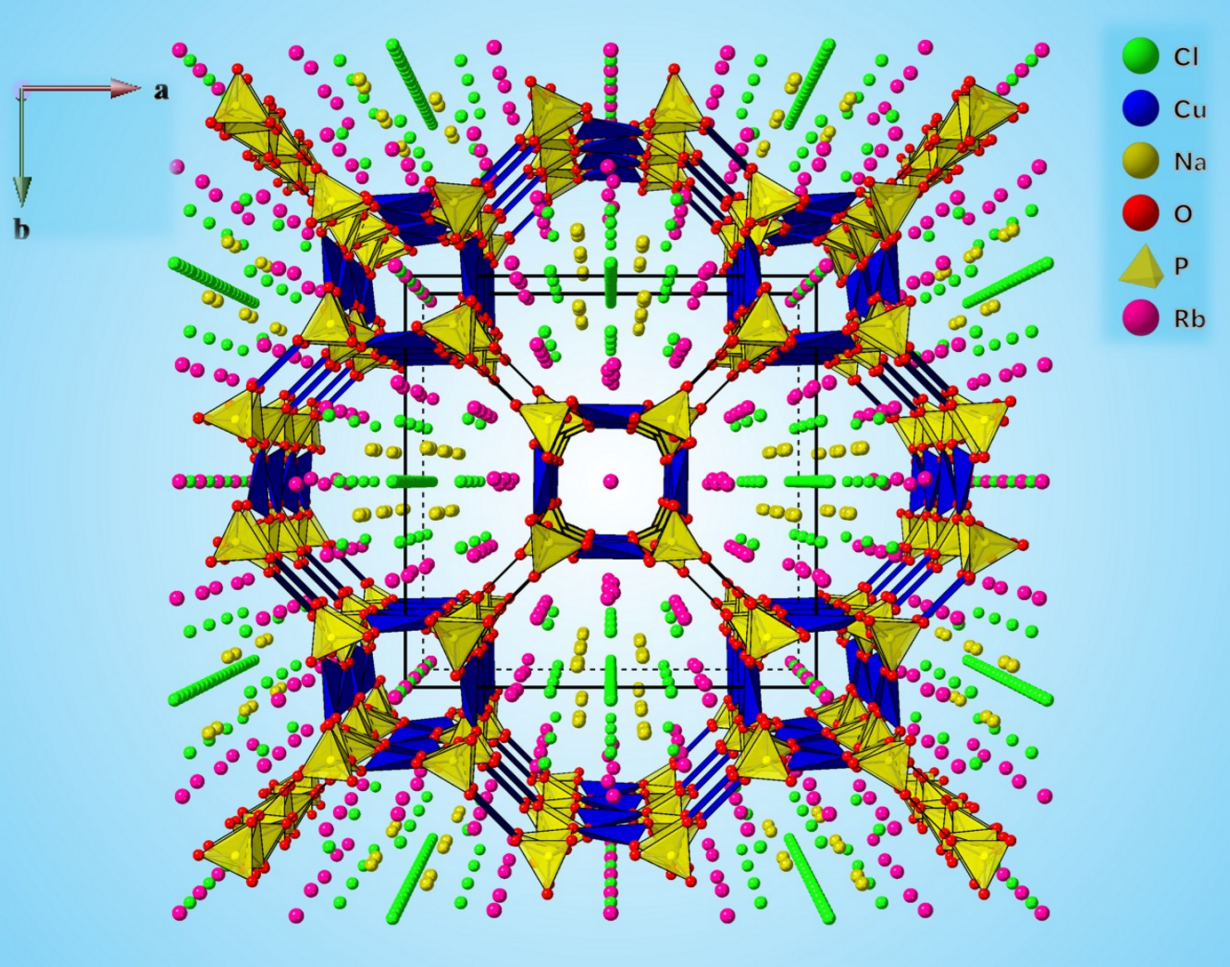
Perspective view of the crystal structure of Rb_9_Na_2_Cu_6_(P_2_O_7_)_4_Cl_7_ along [001]. The alternating P_2_O_7_ and CuO_4_Cl units (given in polyhedral representation; Cu—Cl bonds have been omitted for clarity) are inter­linked through corner-sharing O atoms. The larger channel is occupied by Rb^+^ and Na^+^ cations and Cl^−^ anions, whereas the smaller is occupied by Rb^+^ cations and Cl^−^ anions.

**Figure 2:**
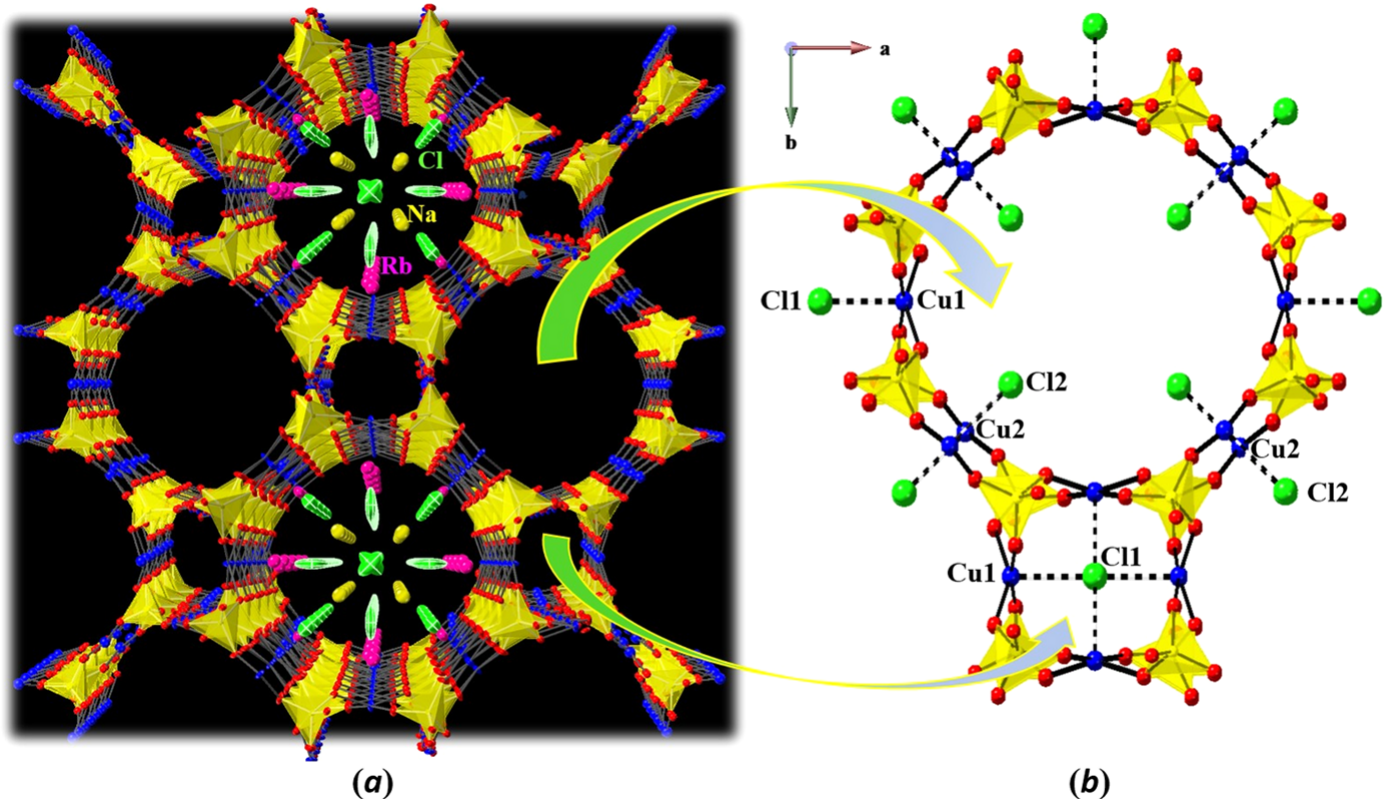
(*a*) The channel structure of Rb_9_Na_2_Cu_6_(P_2_O_7_)_4_Cl_7_ in a view along [001], with the Cu–P–O framework outlined by CuO_4_ and P_2_O_7_ units. The channels are formed by eight (8-ring) or sixteen (16-ring) alternating cations of Cu and P. To indicate the apparent disorder of some atoms (Na, Rb, and Cl), their arrangement is highlighted in two of the large channels. (*b*) Projection of one large and small channel onto (001), with some Cl and all Rb and Na sites are omitted for clarity. Cl atoms occupy the apical position of the Cu^2+^-centered square-pyramidal CuO_4_Cl units (ball-and-stick drawing), with Cu—O bonds as solid lines and Cu—Cl bonds in dotted lines. The polyhedral units represent PO_4_ tetra­hedra.

Rb_9_Na_2_Cu_6_(P_2_O_7_)_4_Cl_7_ crystallizes with four formula units in the space group *I*4/*mcm*. To the best of our knowledge, Rb_9_Na_2_Cu_6_(P_2_O_7_)_4_Cl_7_ represents the fourth member structurally characterized in the CU-2-MXO system (Huang *et al.*, 1999 ▸). The crystal structure of Rb_9_Na_2_Cu_6_(P_2_O_7_)_4_Cl_7_ can be described as having a tri-periodic framework containing channels. As shown in Fig. 1 ▸, the corner-sharing PO_4_ tetra­hedra and CuO_4_ square-planar units form two types of such channels *ca* 5.4 and 11.9 Å in diameter. This is where Rb2 (located at a site with multiplicity 16, Wyckoff letter *l*, symmetry *m*), Rb3 (16*k*, *m*) and Na1 (16*l*, *m*) cations link together with Cl2, Cl3*A*–*D*, and Cl4 anions to reside in the larger channels, while Rb1 (4*c*, 4/*m*) cations and Cl1 (4*a*, 422) anions occupy the smaller channels. As shown in Fig. 2 ▸(*a*), each smaller channel is surrounded by four larger channels and vice versa. The square-planar CuO_4_ units and Cl^−^ ions form CuO_4_Cl square pyramids [Fig. 2 ▸(*b*)]. The open framework is made of the Cu—O—P—O—Cu covalent linkages, leading to alternating CuO_4_Cl units and pyrophosphate [P_2_O_7_] units with a shared vertex O atom O1. It shows that this bridging O1 atom occupies a special site (16*l*, *m*). There are two crystallographically distinct Cu^2+^ sites in the open framework. Cu1 and Cu2 are situated at special sites, 16*j* and 8*h*, with symmetry 2 and *m*2*m*, respectively. The Cl1 atom is common for all the Cu1O_4_Cl units and is occupying the center of the smaller channel, whereas Cl2 (8*h*, *m*2*m*) in Cu2O_4_Cl units face the center of the large channel [Fig. 2 ▸(*b*)].

The oxidation state of the copper cations in the CuO_4_Cl square-pyramidal environment is supported through bond valence sum calculations (Brese & O’Keefe, 1991 ▸), with 2.09 valence units for Cu1 and 2.06 valence units for Cu2. Moreover, all the Cu—O bond lengths (Table 1 ▸) are consistent with what is expected from the sum of the Shannon crystal radii (Shannon, 1976 ▸) for five-coordinate Cu^2+^ and two-coordinate O^2−^ (2.00 Å), whereas the Cu—Cl bond lengths (Table 1 ▸) are somewhat longer than the sum of the Shannon crystal radii (2.46 Å) of five-coordinate Cu^2+^ (0.79 Å) and Cl^−^ (1.67).

**Figure 3:**
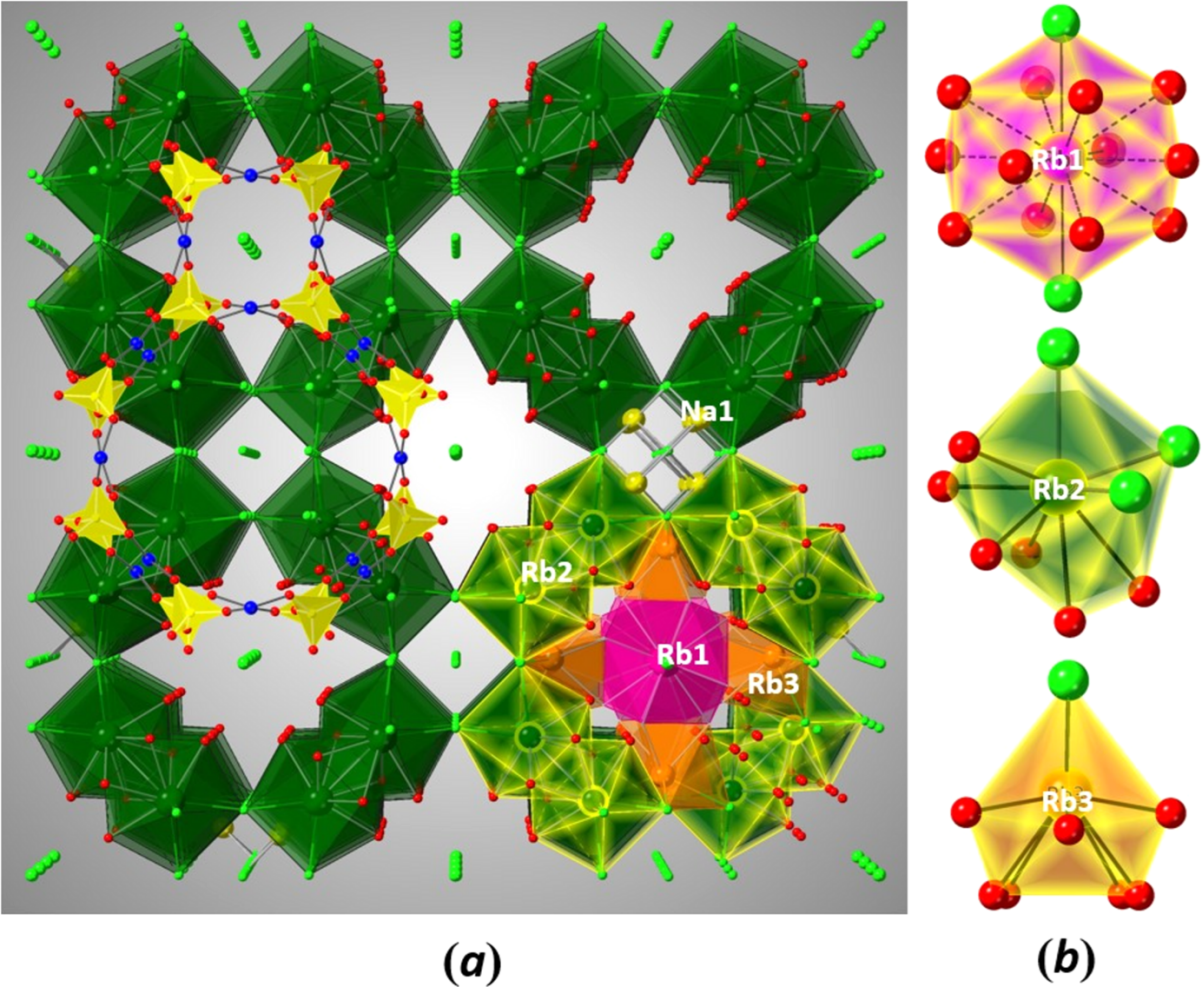
(*a*) Partial structure of Rb_9_Na_2_Cu_6_(P_2_O_7_)_4_Cl_7_ viewed along [001], showing the building blocks of the Rb–O–Cl and NaCl fragments. A portion of the Cu–P–O framework is included to show the location of one large and one small channel. All the Rb_2_O_6_Cl_3_ polyhedra are included, while others are omitted for clarity. The bottom right section shows the actual arrangement of all the Rb–O–Cl polyhedral units and NaCl units. (*b*) The polyhedra representing the coordination around Rb1 (pink), Rb2 (green), and Rb3 (orange).

The P atom is surrounded by four O atoms to form an almost regular tetra­hedron. The terminal P—O bond lengths average to about 1.52 Å, the sum of the Shannon crystal radii (Shannon, 1976 ▸) for P^5+^ (0.31 Å) and O^2−^ (1.21 Å). As expected for a condensed pyrophosphate group (Durif, 1995 ▸), the bond length to the bridging O1 atom is longer (Table 1 ▸). The calculated bond valence sum confirms the oxidation state of P^5+^, *i.e.* 4.79 valence units for P1. Fig. 3 ▸(*a*) shows how the salt-like parts of the structure, Rb^+^, Na^+^, and Cl^−^ ions are linked to the negatively charged wall of the Cu_3_(P_2_O_7_)_2_^2−^ framework. The three crystallographically different rubidium cations form significantly different polyhedra with oxygen and chlorine, as shown in Fig. 3 ▸(*b*).

**Figure 4:**
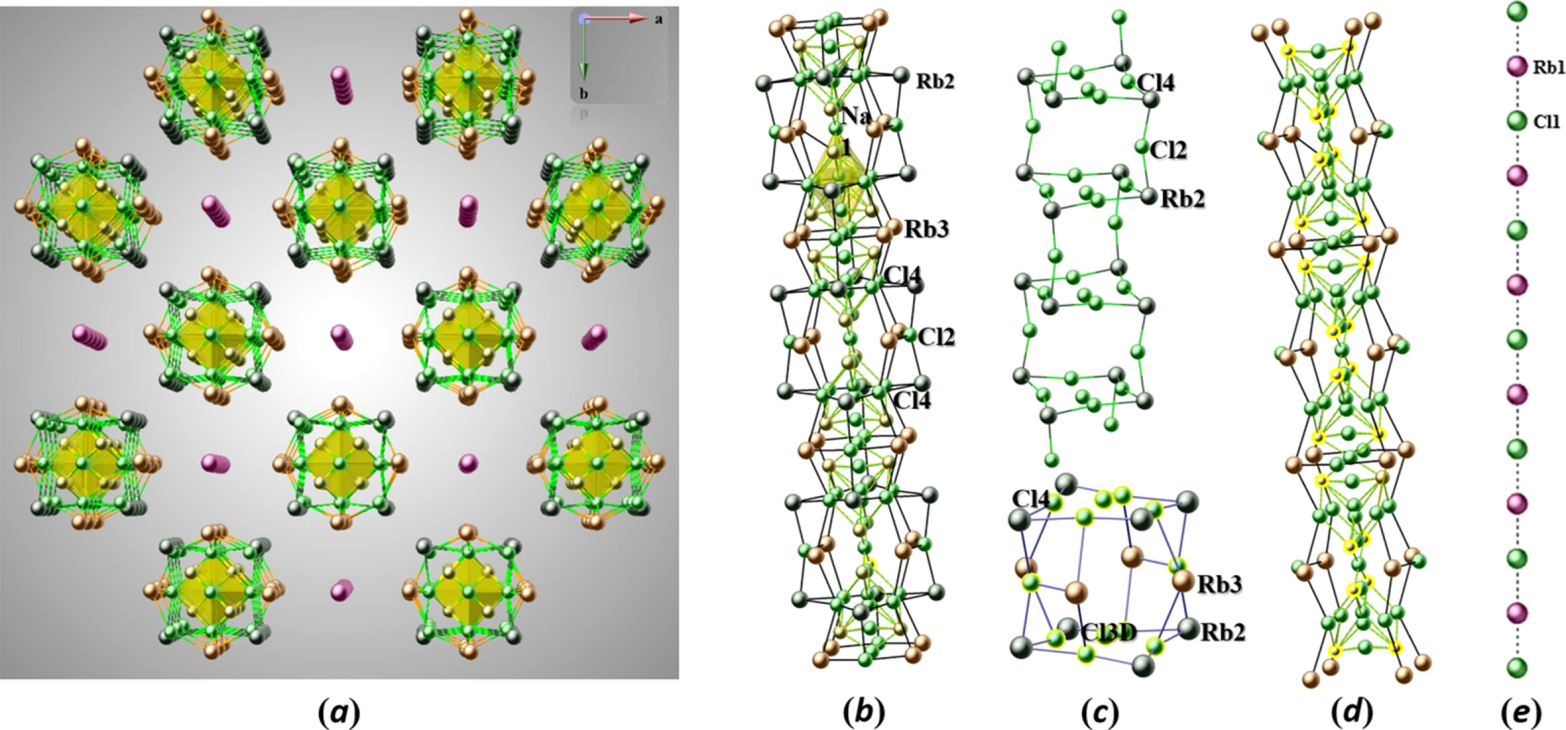
(*a*) Perspective view of the Rb–Cl and Rb–Na–Cl salt-like structural units in a view along [001] (color codes: Rb1 pink, Rb2 dark green, Rb3 orange, Na1 gold, and Cl light green). (*b*) Ball-and-stick drawing of the extended salt structure occupying the larger channel. One Na–Cl unit (see the one in yellow) is highlighted as a polyhedral unit. (*c*) Extended Rb2–Cl salt structural unit forming NaCl-type fragments (top); ‘cubane’-like fragments formed together with Rb3 (bottom), whereby Rb2 occupies all the corners, Rb3 occupies four of the six faces, while Cl3*D* occupies the other two faces, and Cl4 occupies eight of the twelve edges. (*d*) Salt stucture extending along [001], showing the corner-sharing {Na_4_Cl_8_} units (Rb2 excluded for clarity). (*e*) Alternating Rb1 and Cl1 sites in the smaller channel.

**Figure 5:**
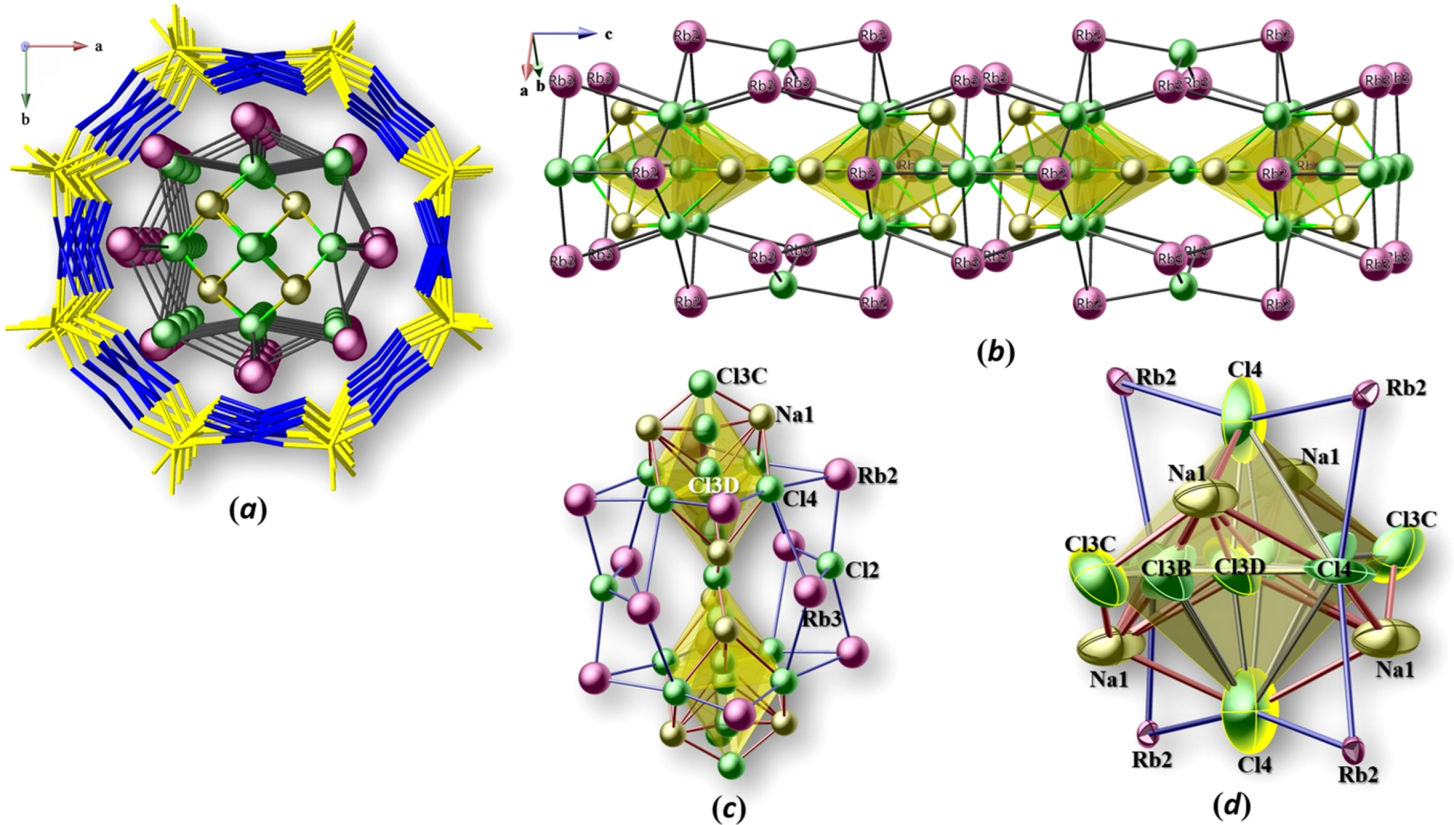
(*a*) Partial structure showing the Rb–Na–Cl salt structure along [001], occupying the larger channel. (*b*) Extended salt structural unit in the larger channel with polyhedral representation showing the corner-sharing {Na_4_Cl_8_} units. (*c*) The arrangement of two corner-sharing {Na_4_Cl_8_} units centered in the faces of a ‘cubane’-like fragment. (*d*) Fragment of the Rb–Na–Cl salt structure, showing the polyhedral arrangement of Cl and Na. Displacement ellipsoids are pre­sent­ed at the 90% probability level.

**Figure 6:**
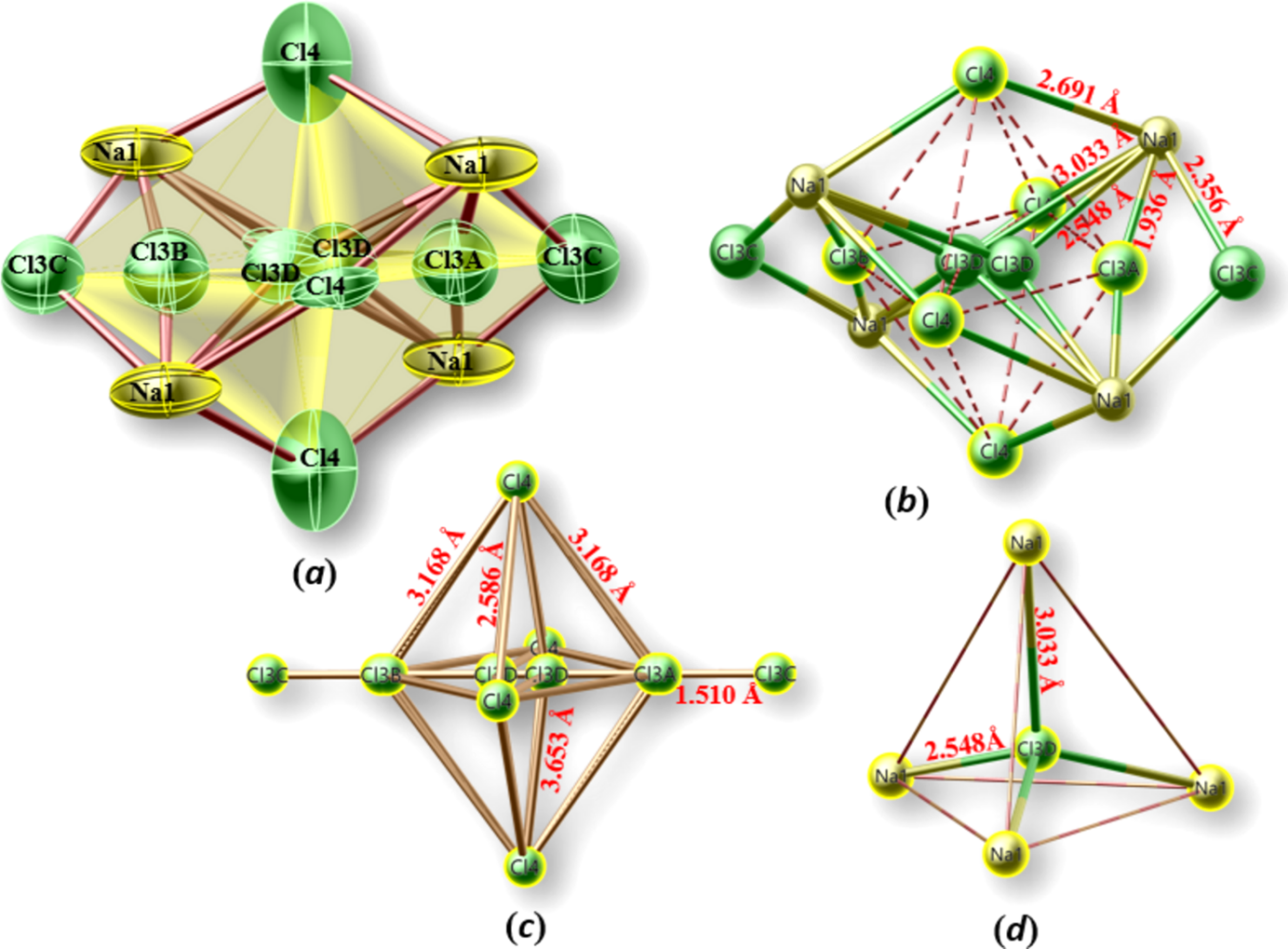
(*a*) Projected view of a {Na_4_Cl_8_} unit [displacement ellipsoids as in Fig. 5 ▸(*d*)]. (*b*) The octa­hedral arrangement of Cl3*A*, Cl3*B*, Cl3*D*, and Cl4 in an {Na_4_Cl_8_} unit. (*c*) Partial structure showing the lengths between some Cl sites. (*d*) μ_4_-Cl3*D*-centered Na4 unit with distorted tetra­hedral shape.

In CU-2-MXO materials (Huang *et al.*., 1999 ▸), the smaller channel is centered by a linear chain-like fragment of alternating *A*–Cl–*A* units (*A* = K, Rb, Cs), while the large channel is stuffed with mixed KCl/CsCl salt-like structure units, which adopt features characteristic for the crystal structures of NaCl and CsCl. Another series of materials adopting the framework of CU-2 topology with general formula *A_x_*Cu_6_(P_2_O_7_)_4_Cl_(*x*-6)_ (Williams *et al.*, 2013 ▸) exhibits a wide variety of com­plex inorganic anions trapped in the large channels, with anions including chloride, bromide, phosphate and the com­plex metal halogenido anions [PtCl_4_]^2−^, [PdBr_4_]^2−^, [CuCl_4_]^2−^, or [AuCl_4_]^−^. Fig. 4 ▸(*a*) shows a perspective view of the Rb–Cl and Rb–Na–Cl salt structural units along [001], and Figs. 4 ▸(*b*)–(*e*) illustrate details of these units in side views. Fig. 5 ▸(*a*) shows the partial structure of the Rb–Na–Cl salt structural unit occupying the larger channel and running along [001]. There is disorder found at the central part of the Rb–Na–Cl salt structure creating partially occupied Na and Cl sites that form corner-sharing {Na_4_Cl_8_} units [Figs. 5 ▸(*b*)–(*d*) and 6)]. Four split sites for Cl3 are present within the {Na_4_Cl_8_} units, Cl3*A* (8*g*, 2*mm*), Cl3*B* (8*g*, 2*mm*), Cl3*C* (4*d*, *mmm*), and Cl3*D* (8*g*, 2*mm*), as well as one Cl4 (16*j*, 2) and one Na1(16*l*, *m*) site with an occupancy of 0.5 each. Cl3*A*, Cl3*B*, Cl3*D*, and Cl4 form an octa­hedron with respect to one another, shown in Fig. 6 ▸(*b*), where half of Cl4 would be in the axial position and the other half of the Cl4, Cl3*A*, and Cl3*B* would be in the four equatorial positions. It is intriguing to recognize the formation of structurally isolated corner-sharing {Na_4_Cl_8_} units that are ‘em­bed­ded’ in the extended rubidium chloride salt structure.

Transition metals and main-group elements form especially robust clusters and their investigation provides valuable insight into how physicochemical properties evolve going from mol­ecular systems to the solid state (Berry, 1993 ▸). Much attention has been paid to studies on sodium chloride clusters using theoretical approaches, especially on the structural shapes and relative stabilities in neutral and charged clusters (Zhang & Chen, 2003 ▸). We hope that the structurally isolated {Na_4_Cl_8_} units in the title com­pound can be relevant for the discussion of the nature of chemical bonding for the theoreticians to apply a simple electrostatic model to describe the energies and stabilities of this sodium chloride unit based on inversion pair potentials. Furthermore, controlling the inter­action between the two chemically dissimilar structural units (halides and oxides) may give rise to new material design by placing efforts on the targeted growth of salt-inclusion com­pounds *via* the careful selection of systems and the use of a mixed alkali halide eutectic flux.

## 3. Synthesis and crystallization

**Figure 7:**
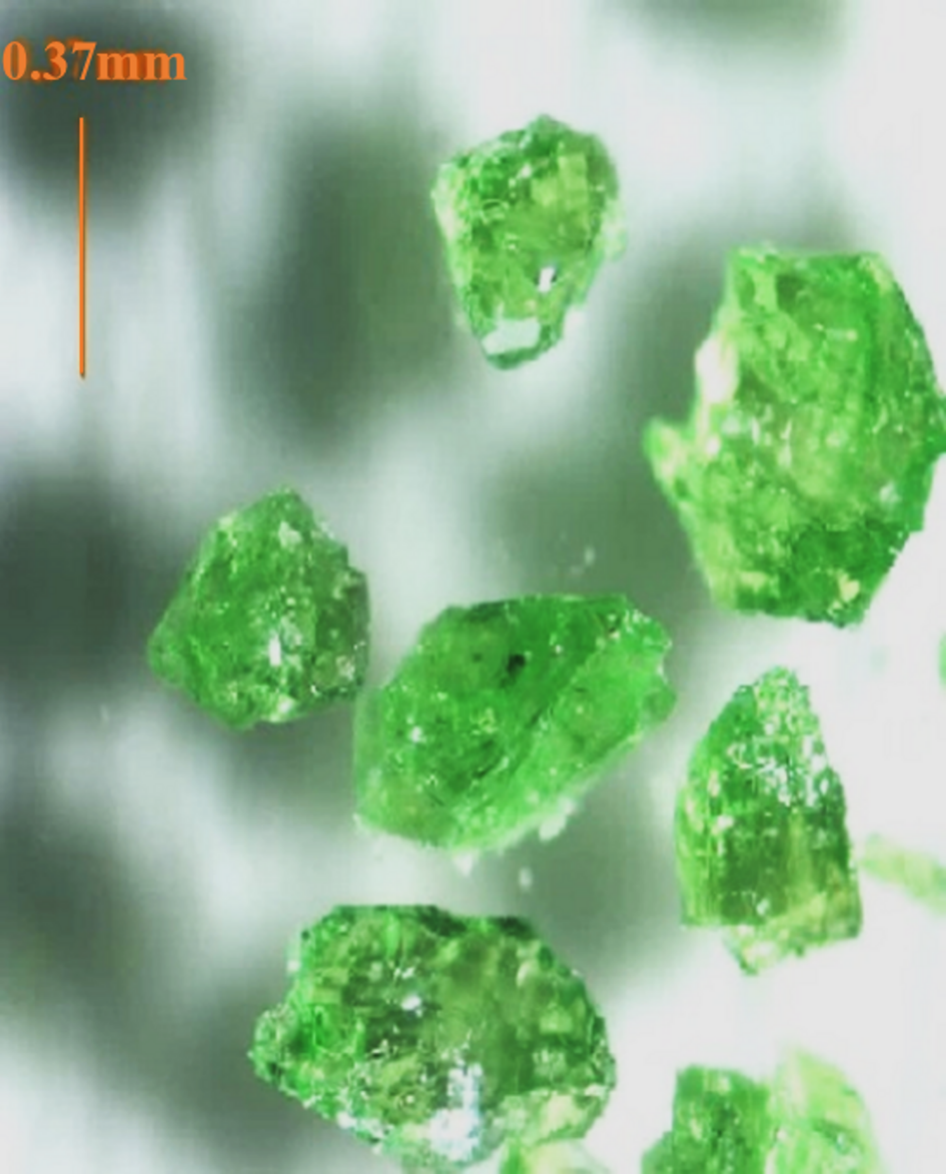
Crystal aggregates of Rb_9_Na_2_Cu_6_(P_2_O_7_)_4_Cl_7_ obtained from a RbCl/NaCl eutectic flux.

Single crystals of Rb_9_Na_2_Cu_6_(P_2_O_7_)_4_Cl_7_ were grown using a eutectic RbCl/NaCl flux in a fused-silica ampoule. The eutectic flux used was 53% RbCl (Alfa, 99.8%) and 47% NaCl (Strem, 99.999%) by moles (melting point 823.4 K). The reactants were ground and loaded in a nitro­gen-blanketed dry box and then sealed under vacuum prior to heating. Crystals were grown by introducing the reactants, *i.e.* P_4_O_10_ (2.1 mmol, Aldrich, 98+%), Rb_2_O (2.1 mmol, Aldrich, 99+%), and CuO (6.3 mmol, Alfa, 99.7%), to the eutectic RbCl/NaCl flux with a flux-to-charge ratio of 3:1. The resulting mixture was loaded into a silica ampoule and the reaction mixture was heated to 923 K at a rate of 2 K min^−1^, dwelled for 2 d and then cooled slowly to 573 K at a rate of 0.1 K min^−1^, followed by cooling to room tem­per­a­ture at a rate of 3 K min^−1^. Irregular-shaped light-green crystals of Rb_9_Na_2_Cu_6_(P_2_O_7_)_4_Cl_7_ (Fig. 7 ▸) were isolated manually and washed with deionized water using suction filtration methods.

## 4. Refinement

Crystal data, data collection and structure refinement details are summarized in Table 2 ▸. The final Fourier difference synthesis showed the maximum residual electron density of 2.37 e Å^−3^ located at 1.82 Å from Na1 and the minimum of −4.12 e Å^−3^ directly at the Cl3*C* position. Refinements were carried out in com­parison with the parent structure of Rb_9_Cu_6_(P_2_O_7_)_4_(CuCl_7_) (Williams *et al.*, 2013 ▸). All positions remained the same up to the point of Cu being exchanged for Na, which, in turn, alters the sites of some Cl atoms as well. The parent structure has the Cu3 atom position at Wyckoff site 4*b* and is fully occupied. When Cu is exchanged by Na on this site, the Na position changes to Wyckoff site 16*l* and becomes half-occupied to maintain charge neutrality. This can be attributed to the larger size of sodium needing to shift slightly off the special position into a general position, which disturbs the channel that contains the chloride anions. In the parent structure, Cl1 is positioned at Wyckoff site 16*j* and corresponds to the disordered Cl3 and Cl4 sites reported herein. Cl3 was split into four sites, Cl3*A*, Cl3*B*, Cl3*C*, and Cl3*D*, with site occupation factors of 0.167, 0.333, 0.667, and 0.167, adding up to half-occupancy for Cl3, which is half of what is reported in the parent structure. To maintain charge neutrality, Cl4 was added at Wyckoff site 16*j* with half-occupancy to obtain charge neutrality. It is important to note that in com­parison to the parent structure, Cl2 and Cl3 correspond to Cl1 and Cl2 within the structure pre­sent­ed herein. The observed minimum electron density noted above indicates that another spliting of the Cl3*C* site might be necessary, but the refinement in this case resulted in models that were not meaningful.

## Supplementary Material

Crystal structure: contains datablock(s) I. DOI: 10.1107/S2056989026004287/wm5786sup1.cif

Structure factors: contains datablock(s) I. DOI: 10.1107/S2056989026004287/wm5786Isup2.hkl

CCDC reference: 2548269

Additional supporting information:  crystallographic information; 3D view; checkCIF report

## Figures and Tables

**Table 1:** Selected bond lengths (Å)

Cu1—O4^i^	1.947 (7)	Cu2—O3^vi^	1.938 (7)
Cu1—O4^ii^	1.947 (7)	Cu2—O3^vii^	1.938 (7)
Cu1—O2^iii^	1.950 (7)	Cu2—Cl2^viii^	2.797 (9)
Cu1—O2^iv^	1.950 (7)	P1—O2	1.507 (7)
Cu1—Cl1	2.7281 (19)	P1—O4	1.516 (7)
Cu2—O3	1.938 (7)	P1—O3^ix^	1.520 (7)
Cu2—O3^v^	1.938 (7)	P1—O1	1.616 (4)

Symmetry codes: (i) 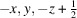; (ii) 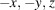; (iii) 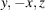; (iv) 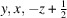; (v) 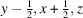; (vi) 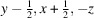; (vii) 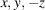; (viii) 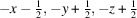; (ix) 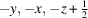.

**Table 2:** Experimental details

Crystal data	
Chemical formula	Rb_9_Na_2_Cu_6_(P_2_O_7_)_4_Cl_7_
*M*_r_	2140.36
Crystal system, space group	Tetragonal, *I*4/*mcm*
Temperature (K)	298
*a*, *c* (Å)	17.840 (3), 13.483 (3)
*V* (Å^3^)	4291.2 (15)
*Z*	4
Radiation type	Mo *K*α
μ (mm^−1^)	13.90
Crystal size (mm)	0.09 × 0.07 × 0.05

Data collection	
Diffractometer	Bruker D8 Quest Photon 3 CCD
Absorption correction	Multi-scan (*SADABS*; Krause *et al.*, 2015 ▸)
*T*_min_, *T*_max_	0.849, 1.000
No. of measured, independent and observed [*I* > 2σ(*I*)] reflections	18814, 1105, 1048
*R*_int_	0.058
(sin θ/λ)_max_ (Å^−1^)	0.606

Refinement	
*R*[*F*^2^ > 2σ(*F*^2^)], *wR*(*F*^2^), *S*	0.063, 0.164, 1.12
No. of reflections	1105
No. of parameters	98
No. of restraints	30
Δρ_max_, Δρ_min_ (e Å^−3^)	2.37, −4.12

Computer programs: *APEX3* and *SAINT* (Bruker, 2017 ▸), *SHELXT* (Sheldrick, 2015*a* ▸), *SHELXL* (Sheldrick, 2015*b* ▸), *CrystalMaker* (Palmer, 2014 ▸) and *publCIF* (Westrip, 2010 ▸).

## supplementary crystallographic information

### Nonarubidium disodium hexacopper(II) tetrakis(pyrophosphate) heptachloride Crystal data

**Table:**

Rb_9_Na_2_Cu_6_(P_2_O_7_)_4_Cl_7_	*D*_x_ = 3.313 Mg m^−^^3^
*M**_r_* = 2140.36	Mo *K*α radiation, λ = 0.71073 Å
Tetragonal, *I*4/*m**c**m*	Cell parameters from 6572 reflections
*a* = 17.840 (3) Å	θ = 3.2–26.9°
*c* = 13.483 (3) Å	µ = 13.90 mm^−^^1^
*V* = 4291.2 (15) Å^3^	*T* = 298 K
*Z* = 4	Block, green
*F*(000) = 3968	0.09 × 0.07 × 0.05 mm

### Nonarubidium disodium hexacopper(II) tetrakis(pyrophosphate) heptachloride Data collection

**Table:**

Bruker D8 Quest Photon 3 CCD diffractometer	1048 reflections with *I* > 2σ(*I*)
Radiation source: Incoatec IµS	*R*_int_ = 0.058
φ and ω scans	θ_max_ = 25.5°, θ_min_ = 2.3°
Absorption correction: multi-scan (*SADABS*; Krause *et al.*, 2015)	*h* = −21→21
*T*_min_ = 0.849, *T*_max_ = 1.000	*k* = −21→21
18814 measured reflections	*l* = −16→16
1105 independent reflections	

### Nonarubidium disodium hexacopper(II) tetrakis(pyrophosphate) heptachloride Refinement

**Table:**

Refinement on *F*^2^	98 parameters
Least-squares matrix: full	30 restraints
*R*[*F*^2^ > 2σ(*F*^2^)] = 0.063	*w* = 1/[σ^2^(*F*_o_^2^) + (0.0661*P*)^2^ + 335.4492*P*] where *P* = (*F*_o_^2^ + 2*F*_c_^2^)/3
*wR*(*F*^2^) = 0.164	(Δ/σ)_max_ < 0.001
*S* = 1.12	Δρ_max_ = 2.37 e Å^−^^3^
1105 reflections	Δρ_min_ = −4.12 e Å^−^^3^

### Nonarubidium disodium hexacopper(II) tetrakis(pyrophosphate) heptachloride Special details

**Table:**

Geometry. All esds (except the esd in the dihedral angle between two l.s. planes) are estimated using the full covariance matrix. The cell esds are taken into account individually in the estimation of esds in distances, angles and torsion angles; correlations between esds in cell parameters are only used when they are defined by crystal symmetry. An approximate (isotropic) treatment of cell esds is used for estimating esds involving l.s. planes.

### Nonarubidium disodium hexacopper(II) tetrakis(pyrophosphate) heptachloride Fractional atomic coordinates and isotropic or equivalent isotropic displacement parameters (Å^2^)

**Table:**

	*x*	*y*	*z*	*U*_iso_*/*U*_eq_	Occ. (<1)
Rb1	0.000000	0.000000	0.000000	0.0186 (6)	
Rb2	−0.17521 (7)	0.32479 (7)	0.26679 (11)	0.0245 (4)	
Rb3	0.00646 (12)	0.27062 (11)	0.500000	0.0417 (6)	
Cu1	0.15292 (11)	0.000000	0.250000	0.0137 (4)	
Cu2	−0.23588 (10)	0.26412 (10)	0.000000	0.0119 (6)	
P1	−0.14115 (13)	0.13979 (13)	0.39130 (16)	0.0065 (5)	
O1	−0.1038 (5)	0.1476 (5)	0.500000	0.0084 (18)	
O2	−0.0891 (4)	0.1826 (4)	0.3239 (5)	0.0121 (14)	
O3	−0.1760 (4)	0.2182 (4)	0.1034 (5)	0.0164 (16)	
O4	−0.1452 (4)	0.0559 (4)	0.3735 (5)	0.0151 (15)	
Cl1	0.000000	0.000000	0.250000	0.0250 (16)	
Cl2	−0.1533 (4)	0.3467 (4)	0.500000	0.0455 (18)	
Cl3A	0.500000	0.000000	0.118 (11)	0.107 (8)	0.1667
Cl3D	0.500000	0.000000	0.275 (6)	0.110 (8)	0.1667
Cl3B	0.500000	0.000000	0.388 (6)	0.108 (8)	0.3333
Cl3C	0.500000	0.000000	0.500000	0.118 (8)	0.6666
Cl4	0.3563 (17)	0.000000	0.250000	0.135 (10)	0.5
Na1	0.5772 (9)	0.0772 (9)	0.399 (3)	0.102 (11)	0.5

### Nonarubidium disodium hexacopper(II) tetrakis(pyrophosphate) heptachloride Atomic displacement parameters (Å^2^)

**Table:**

	*U*^11^	*U*^22^	*U*^33^	*U*^12^	*U*^13^	*U*^23^
Rb1	0.0196 (9)	0.0196 (9)	0.0166 (13)	0.000	0.000	0.000
Rb2	0.0275 (6)	0.0275 (6)	0.0186 (8)	0.0074 (6)	−0.0047 (5)	−0.0047 (5)
Rb3	0.0376 (11)	0.0346 (10)	0.0528 (12)	0.0058 (8)	0.000	0.000
Cu1	0.0295 (10)	0.0083 (8)	0.0033 (7)	0.000	0.000	0.0002 (6)
Cu2	0.0168 (8)	0.0168 (8)	0.0020 (10)	0.0113 (10)	0.000	0.000
P1	0.0089 (12)	0.0081 (12)	0.0024 (10)	0.0024 (8)	0.0001 (9)	−0.0002 (9)
O1	0.010 (5)	0.013 (5)	0.002 (4)	0.002 (4)	0.000	0.000
O2	0.014 (3)	0.013 (3)	0.009 (3)	0.004 (3)	0.001 (3)	0.003 (3)
O3	0.033 (4)	0.011 (3)	0.005 (3)	0.010 (3)	−0.001 (3)	−0.002 (3)
O4	0.027 (4)	0.011 (3)	0.007 (3)	−0.002 (3)	−0.004 (3)	−0.003 (3)
Cl1	0.024 (2)	0.024 (2)	0.027 (4)	0.000	0.000	0.000
Cl2	0.058 (3)	0.058 (3)	0.020 (3)	0.014 (4)	0.000	0.000
Cl3A	0.107 (10)	0.107 (10)	0.106 (16)	−0.082 (16)	0.000	0.000
Cl3D	0.110 (10)	0.110 (10)	0.109 (15)	−0.080 (15)	0.000	0.000
Cl3B	0.108 (10)	0.108 (10)	0.106 (15)	−0.083 (14)	0.000	0.000
Cl3C	0.120 (10)	0.120 (10)	0.115 (15)	−0.072 (14)	0.000	0.000
Cl4	0.25 (3)	0.025 (6)	0.130 (17)	0.000	0.000	−0.021 (9)
Na1	0.047 (7)	0.047 (7)	0.21 (3)	0.006 (9)	0.006 (12)	0.006 (12)

### Nonarubidium disodium hexacopper(II) tetrakis(pyrophosphate) heptachloride Geometric parameters (Å, º)

**Table:**

Rb1—O1^i^	3.220 (9)	Cu1—O4^xx^	1.947 (7)
Rb1—O1^ii^	3.220 (9)	Cu1—O2^xiv^	1.950 (7)
Rb1—O1^iii^	3.220 (9)	Cu1—O2^iv^	1.950 (7)
Rb1—O1^iv^	3.220 (9)	Cu1—Cl1	2.7281 (19)
Rb1—O4^i^	3.257 (7)	Cu2—O3	1.938 (7)
Rb1—O4^ii^	3.257 (7)	Cu2—O3^ix^	1.938 (7)
Rb1—O4^v^	3.257 (7)	Cu2—O3^xxi^	1.938 (7)
Rb1—O4^vi^	3.257 (7)	Cu2—O3^xxii^	1.938 (7)
Rb1—O4^vii^	3.257 (7)	Cu2—Cl2^xii^	2.797 (9)
Rb1—O4^viii^	3.257 (7)	P1—O2	1.507 (7)
Rb1—O4^iii^	3.257 (7)	P1—O4	1.516 (7)
Rb1—O4^iv^	3.257 (7)	P1—O3^vi^	1.520 (7)
Rb2—O3	2.911 (7)	P1—O1	1.616 (4)
Rb2—O3^ix^	2.911 (7)	Cl3A—Cl3B^xxiii^	0.1 (2)
Rb2—O2	3.064 (6)	Cl3A—Cl3D^xxiii^	1.43 (17)
Rb2—O2^ix^	3.064 (6)	Cl3A—Cl3C^xxiv^	1.60 (15)
Rb2—Cl4^x^	3.184 (6)	Cl3A—Na1^xxv^	1.96 (3)
Rb2—Cl4^xi^	3.184 (6)	Cl3A—Na1^xxiii^	1.96 (3)
Rb2—Cl2	3.193 (2)	Cl3A—Cl3D	2.12 (17)
Rb2—O3^vi^	3.271 (8)	Cl3D—Cl3D^xxiii^	0.69 (16)
Rb2—O3^xii^	3.271 (8)	Cl3D—Cl3B	1.52 (11)
Rb2—Na1^xiii^	3.33 (3)	Cl3D—Cl3B^xxiii^	2.21 (11)
Rb2—P1	3.752 (3)	Cl3D—Na1^xxvi^	2.56 (6)
Rb2—P1^ix^	3.752 (3)	Cl3D—Na1	2.56 (6)
Rb3—O1	2.947 (10)	Cl3D—Na1^xxv^	3.05 (7)
Rb3—O4^xiv^	2.949 (7)	Cl3D—Na1^xxiii^	3.05 (7)
Rb3—O4^xv^	2.949 (7)	Cl3B—Cl3C	1.51 (7)
Rb3—Cl2	3.156 (4)	Cl3B—Na1^xxvi^	1.95 (2)
Rb3—Na1^xvi^	3.289 (18)	Cl3B—Na1	1.95 (2)
Rb3—Na1^xvii^	3.289 (18)	Cl3C—Na1^xviii^	2.38 (3)
Rb3—O2^xviii^	3.318 (7)	Cl3C—Na1^xxvi^	2.38 (3)
Rb3—O2	3.318 (7)	Cl3C—Na1^xxvii^	2.38 (3)
Rb3—O3^xix^	3.459 (8)	Cl3C—Na1	2.38 (3)
Rb3—O3^ii^	3.459 (8)	Cl4—Na1^xxvi^	2.71 (3)
Rb3—P1^xv^	3.625 (3)	Cl4—Na1^xxv^	2.71 (3)
Rb3—P1^xiv^	3.625 (3)	Na1—Na1^xviii^	2.72 (8)
Cu1—O4^ii^	1.947 (7)	Na1—Na1^xxvi^	3.90 (5)
			
O1^i^—Rb1—O1^ii^	180.0 (3)	O3^vi^—P1—Rb2	60.1 (3)
O1^i^—Rb1—O1^iii^	90.0	O1—P1—Rb2	113.3 (3)
O1^ii^—Rb1—O1^iii^	90.0	Rb3^x^—P1—Rb2	130.91 (8)
O1^i^—Rb1—O1^iv^	90.0	O2—P1—Rb3	59.7 (3)
O1^ii^—Rb1—O1^iv^	90.0	O4—P1—Rb3	134.2 (3)
O1^iii^—Rb1—O1^iv^	180.0 (3)	O3^vi^—P1—Rb3	110.2 (3)
O1^i^—Rb1—O4^i^	44.93 (16)	O1—P1—Rb3	46.6 (3)
O1^ii^—Rb1—O4^i^	135.07 (16)	Rb3^x^—P1—Rb3	133.35 (7)
O1^iii^—Rb1—O4^i^	61.70 (18)	Rb2—P1—Rb3	75.25 (6)
O1^iv^—Rb1—O4^i^	118.30 (18)	O2—P1—Rb1^ii^	99.0 (3)
O1^i^—Rb1—O4^ii^	135.07 (16)	O4—P1—Rb1^ii^	56.6 (3)
O1^ii^—Rb1—O4^ii^	44.93 (16)	O3^vi^—P1—Rb1^ii^	148.4 (3)
O1^iii^—Rb1—O4^ii^	118.30 (18)	O1—P1—Rb1^ii^	55.9 (3)
O1^iv^—Rb1—O4^ii^	61.70 (18)	Rb3^x^—P1—Rb1^ii^	80.63 (5)
O4^i^—Rb1—O4^ii^	180.0 (3)	Rb2—P1—Rb1^ii^	148.13 (8)
O1^i^—Rb1—O4^v^	61.70 (18)	Rb3—P1—Rb1^ii^	78.33 (5)
O1^ii^—Rb1—O4^v^	118.30 (18)	O2—P1—Rb2^xii^	96.0 (3)
O1^iii^—Rb1—O4^v^	135.07 (16)	O4—P1—Rb2^xii^	91.9 (3)
O1^iv^—Rb1—O4^v^	44.93 (16)	O3^vi^—P1—Rb2^xii^	37.8 (3)
O4^i^—Rb1—O4^v^	74.10 (11)	O1—P1—Rb2^xii^	144.6 (3)
O4^ii^—Rb1—O4^v^	105.90 (11)	Rb3^x^—P1—Rb2^xii^	78.18 (6)
O1^i^—Rb1—O4^vi^	118.30 (18)	Rb2—P1—Rb2^xii^	58.99 (5)
O1^ii^—Rb1—O4^vi^	61.70 (18)	Rb3—P1—Rb2^xii^	132.91 (7)
O1^iii^—Rb1—O4^vi^	44.93 (16)	Rb1^ii^—P1—Rb2^xii^	148.52 (7)
O1^iv^—Rb1—O4^vi^	135.07 (16)	P1^xviii^—O1—P1	130.2 (6)
O4^i^—Rb1—O4^vi^	105.90 (11)	P1^xviii^—O1—Rb3	109.9 (3)
O4^ii^—Rb1—O4^vi^	74.10 (11)	P1—O1—Rb3	109.9 (3)
O4^v^—Rb1—O4^vi^	180.0 (3)	P1^xviii^—O1—Rb1^ii^	99.6 (3)
O1^i^—Rb1—O4^vii^	135.07 (16)	P1—O1—Rb1^ii^	99.6 (3)
O1^ii^—Rb1—O4^vii^	44.93 (16)	Rb3—O1—Rb1^ii^	103.0 (3)
O1^iii^—Rb1—O4^vii^	118.30 (18)	P1—O2—Cu1^x^	132.3 (4)
O1^iv^—Rb1—O4^vii^	61.70 (18)	P1—O2—Rb2	105.2 (3)
O4^i^—Rb1—O4^vii^	116.9 (2)	Cu1^x^—O2—Rb2	120.3 (3)
O4^ii^—Rb1—O4^vii^	63.1 (2)	P1—O2—Rb3	97.2 (3)
O4^v^—Rb1—O4^vii^	74.10 (11)	Cu1^x^—O2—Rb3	94.3 (2)
O4^vi^—Rb1—O4^vii^	105.90 (11)	Rb2—O2—Rb3	92.62 (18)
O1^i^—Rb1—O4^viii^	44.93 (16)	P1^vi^—O3—Cu2	130.6 (4)
O1^ii^—Rb1—O4^viii^	135.07 (16)	P1^vi^—O3—Rb2	123.6 (4)
O1^iii^—Rb1—O4^viii^	61.70 (18)	Cu2—O3—Rb2	105.7 (3)
O1^iv^—Rb1—O4^viii^	118.30 (18)	P1^vi^—O3—Rb2^xii^	96.2 (3)
O4^i^—Rb1—O4^viii^	63.1 (2)	Cu2—O3—Rb2^xii^	92.1 (3)
O4^ii^—Rb1—O4^viii^	116.9 (2)	Rb2—O3—Rb2^xii^	75.63 (17)
O4^v^—Rb1—O4^viii^	105.90 (11)	P1^vi^—O3—Rb3^ii^	83.8 (3)
O4^vi^—Rb1—O4^viii^	74.10 (11)	Cu2—O3—Rb3^ii^	94.5 (3)
O4^vii^—Rb1—O4^viii^	180.0 (3)	Rb2—O3—Rb3^ii^	97.1 (2)
O1^i^—Rb1—O4^iii^	118.30 (18)	Rb2^xii^—O3—Rb3^ii^	171.3 (2)
O1^ii^—Rb1—O4^iii^	61.70 (18)	P1—O4—Cu1^xx^	130.1 (4)
O1^iii^—Rb1—O4^iii^	44.93 (16)	P1—O4—Rb3^x^	103.9 (4)
O1^iv^—Rb1—O4^iii^	135.07 (16)	Cu1^xx^—O4—Rb3^x^	106.7 (3)
O4^i^—Rb1—O4^iii^	74.10 (11)	P1—O4—Rb1^ii^	100.5 (3)
O4^ii^—Rb1—O4^iii^	105.90 (11)	Cu1^xx^—O4—Rb1^ii^	110.3 (3)
O4^v^—Rb1—O4^iii^	116.9 (2)	Rb3^x^—O4—Rb1^ii^	102.07 (19)
O4^vi^—Rb1—O4^iii^	63.1 (2)	Cu1—Cl1—Cu1^xiv^	90.0
O4^vii^—Rb1—O4^iii^	74.10 (11)	Cu1—Cl1—Cu1^x^	90.0
O4^viii^—Rb1—O4^iii^	105.90 (11)	Cu1^xiv^—Cl1—Cu1^x^	180.0
O1^i^—Rb1—O4^iv^	61.70 (18)	Cu1—Cl1—Cu1^xx^	180.0
O1^ii^—Rb1—O4^iv^	118.30 (18)	Cu1^xiv^—Cl1—Cu1^xx^	90.0
O1^iii^—Rb1—O4^iv^	135.07 (16)	Cu1^x^—Cl1—Cu1^xx^	90.0
O1^iv^—Rb1—O4^iv^	44.93 (16)	Cu1—Cl1—Rb1	90.0
O4^i^—Rb1—O4^iv^	105.90 (11)	Cu1^xiv^—Cl1—Rb1	90.0
O4^ii^—Rb1—O4^iv^	74.10 (11)	Cu1^x^—Cl1—Rb1	90.0
O4^v^—Rb1—O4^iv^	63.1 (2)	Cu1^xx^—Cl1—Rb1	90.0
O4^vi^—Rb1—O4^iv^	116.9 (2)	Cu1—Cl1—Rb1^ii^	90.0
O4^vii^—Rb1—O4^iv^	105.90 (11)	Cu1^xiv^—Cl1—Rb1^ii^	90.0
O4^viii^—Rb1—O4^iv^	74.10 (11)	Cu1^x^—Cl1—Rb1^ii^	90.0
O4^iii^—Rb1—O4^iv^	180.0 (3)	Cu1^xx^—Cl1—Rb1^ii^	90.0
O3—Rb2—O3^ix^	54.6 (3)	Rb1—Cl1—Rb1^ii^	180.0
O3—Rb2—O2	69.62 (18)	Rb3^xxviii^—Cl2—Rb3	141.0 (3)
O3^ix^—Rb2—O2	120.9 (2)	Rb3^xxviii^—Cl2—Rb2	93.32 (3)
O3—Rb2—O2^ix^	120.9 (2)	Rb3—Cl2—Rb2	93.32 (3)
O3^ix^—Rb2—O2^ix^	69.62 (18)	Rb3^xxviii^—Cl2—Rb2^xviii^	93.32 (3)
O2—Rb2—O2^ix^	140.1 (3)	Rb3—Cl2—Rb2^xviii^	93.32 (3)
O3—Rb2—Cl4^x^	93.8 (4)	Rb2—Cl2—Rb2^xviii^	160.0 (3)
O3^ix^—Rb2—Cl4^x^	126.05 (16)	Cl3B^xxiii^—Cl3A—Cl3D^xxiii^	180.00 (4)
O2—Rb2—Cl4^x^	70.8 (5)	Cl3B^xxiii^—Cl3A—Cl3C^xxiv^	0.00 (2)
O2^ix^—Rb2—Cl4^x^	137.9 (5)	Cl3D^xxiii^—Cl3A—Cl3C^xxiv^	180.0
O3—Rb2—Cl4^xi^	126.05 (16)	Cl3B^xxiii^—Cl3A—Na1^xxv^	83 (4)
O3^ix^—Rb2—Cl4^xi^	93.8 (4)	Cl3D^xxiii^—Cl3A—Na1^xxv^	97 (4)
O2—Rb2—Cl4^xi^	137.9 (5)	Cl3C^xxiv^—Cl3A—Na1^xxv^	83 (4)
O2^ix^—Rb2—Cl4^xi^	70.8 (5)	Cl3B^xxiii^—Cl3A—Na1^xxiii^	83 (4)
Cl4^x^—Rb2—Cl4^xi^	69.4 (11)	Cl3D^xxiii^—Cl3A—Na1^xxiii^	97 (4)
O3—Rb2—Cl2	145.72 (17)	Cl3C^xxiv^—Cl3A—Na1^xxiii^	83 (4)
O3^ix^—Rb2—Cl2	145.71 (17)	Na1^xxv^—Cl3A—Na1^xxiii^	166 (9)
O2—Rb2—Cl2	78.02 (13)	Cl3B^xxiii^—Cl3A—Cl3D	180.00 (3)
O2^ix^—Rb2—Cl2	78.02 (13)	Cl3D^xxiii^—Cl3A—Cl3D	0.000 (3)
Cl4^x^—Rb2—Cl2	85.87 (15)	Cl3C^xxiv^—Cl3A—Cl3D	180.0
Cl4^xi^—Rb2—Cl2	85.87 (15)	Na1^xxv^—Cl3A—Cl3D	97 (4)
O3—Rb2—O3^vi^	82.8 (2)	Na1^xxiii^—Cl3A—Cl3D	97 (4)
O3^ix^—Rb2—O3^vi^	104.37 (17)	Cl3D^xxiii^—Cl3D—Cl3A^xxiii^	180.00 (2)
O2—Rb2—O3^vi^	46.45 (17)	Cl3D^xxiii^—Cl3D—Cl3B	180.000 (12)
O2^ix^—Rb2—O3^vi^	94.48 (18)	Cl3A^xxiii^—Cl3D—Cl3B	0.000 (11)
Cl4^x^—Rb2—O3^vi^	114.3 (5)	Cl3D^xxiii^—Cl3D—Cl3A	0.000 (13)
Cl4^xi^—Rb2—O3^vi^	151.18 (16)	Cl3A^xxiii^—Cl3D—Cl3A	180.0
Cl2—Rb2—O3^vi^	66.49 (19)	Cl3B—Cl3D—Cl3A	180.000 (16)
O3—Rb2—O3^xii^	104.37 (17)	Cl3D^xxiii^—Cl3D—Cl3B^xxiii^	0.000 (10)
O3^ix^—Rb2—O3^xii^	82.8 (2)	Cl3A^xxiii^—Cl3D—Cl3B^xxiii^	180.000 (16)
O2—Rb2—O3^xii^	94.48 (18)	Cl3B—Cl3D—Cl3B^xxiii^	180.0
O2^ix^—Rb2—O3^xii^	46.45 (17)	Cl3A—Cl3D—Cl3B^xxiii^	0.000 (3)
Cl4^x^—Rb2—O3^xii^	151.18 (16)	Cl3D^xxiii^—Cl3D—Na1^xxvi^	130.5 (16)
Cl4^xi^—Rb2—O3^xii^	114.3 (5)	Cl3A^xxiii^—Cl3D—Na1^xxvi^	49.5 (16)
Cl2—Rb2—O3^xii^	66.49 (19)	Cl3B—Cl3D—Na1^xxvi^	49.5 (16)
O3^vi^—Rb2—O3^xii^	48.2 (2)	Cl3A—Cl3D—Na1^xxvi^	130.5 (16)
O3—Rb2—Na1^xiii^	80.6 (5)	Cl3B^xxiii^—Cl3D—Na1^xxvi^	130.5 (16)
O3^ix^—Rb2—Na1^xiii^	80.6 (5)	Cl3D^xxiii^—Cl3D—Na1	130.5 (16)
O2—Rb2—Na1^xiii^	109.88 (13)	Cl3A^xxiii^—Cl3D—Na1	49.5 (16)
O2^ix^—Rb2—Na1^xiii^	109.88 (13)	Cl3B—Cl3D—Na1	49.5 (16)
Cl4^x^—Rb2—Na1^xiii^	49.1 (5)	Cl3A—Cl3D—Na1	130.5 (16)
Cl4^xi^—Rb2—Na1^xiii^	49.1 (5)	Cl3B^xxiii^—Cl3D—Na1	130.5 (16)
Cl2—Rb2—Na1^xiii^	122.1 (6)	Na1^xxvi^—Cl3D—Na1	99 (3)
O3^vi^—Rb2—Na1^xiii^	155.17 (18)	Cl3D^xxiii^—Cl3D—Na1^xxv^	39.6 (11)
O3^xii^—Rb2—Na1^xiii^	155.17 (18)	Cl3A^xxiii^—Cl3D—Na1^xxv^	140.4 (11)
O3—Rb2—P1	76.40 (14)	Cl3B—Cl3D—Na1^xxv^	140.4 (11)
O3^ix^—Rb2—P1	116.12 (17)	Cl3A—Cl3D—Na1^xxv^	39.6 (11)
O2—Rb2—P1	22.79 (13)	Cl3B^xxiii^—Cl3D—Na1^xxv^	39.6 (11)
O2^ix^—Rb2—P1	117.55 (14)	Na1^xxvi^—Cl3D—Na1^xxv^	120.0 (9)
Cl4^x^—Rb2—P1	91.6 (5)	Na1—Cl3D—Na1^xxv^	120.0 (9)
Cl4^xi^—Rb2—P1	150.1 (3)	Cl3D^xxiii^—Cl3D—Na1^xxiii^	39.6 (11)
Cl2—Rb2—P1	69.35 (11)	Cl3A^xxiii^—Cl3D—Na1^xxiii^	140.4 (11)
O3^vi^—Rb2—P1	23.75 (12)	Cl3B—Cl3D—Na1^xxiii^	140.4 (11)
O3^xii^—Rb2—P1	71.69 (13)	Cl3A—Cl3D—Na1^xxiii^	39.6 (11)
Na1^xiii^—Rb2—P1	132.57 (4)	Cl3B^xxiii^—Cl3D—Na1^xxiii^	39.6 (11)
O3—Rb2—P1^ix^	116.12 (17)	Na1^xxvi^—Cl3D—Na1^xxiii^	120.0 (9)
O3^ix^—Rb2—P1^ix^	76.40 (14)	Na1—Cl3D—Na1^xxiii^	120.0 (9)
O2—Rb2—P1^ix^	117.55 (14)	Na1^xxv^—Cl3D—Na1^xxiii^	79 (2)
O2^ix^—Rb2—P1^ix^	22.79 (13)	Cl3A^xxiii^—Cl3B—Cl3C	180.00 (19)
Cl4^x^—Rb2—P1^ix^	150.1 (3)	Cl3A^xxiii^—Cl3B—Cl3D	0.0 (2)
Cl4^xi^—Rb2—P1^ix^	91.6 (5)	Cl3C—Cl3B—Cl3D	180.000 (12)
Cl2—Rb2—P1^ix^	69.35 (11)	Cl3A^xxiii^—Cl3B—Na1^xxvi^	94 (3)
O3^vi^—Rb2—P1^ix^	71.69 (13)	Cl3C—Cl3B—Na1^xxvi^	86 (2)
O3^xii^—Rb2—P1^ix^	23.75 (12)	Cl3D—Cl3B—Na1^xxvi^	94 (2)
Na1^xiii^—Rb2—P1^ix^	132.57 (4)	Cl3A^xxiii^—Cl3B—Na1	94 (2)
P1—Rb2—P1^ix^	94.86 (8)	Cl3C—Cl3B—Na1	86 (2)
O1—Rb3—O4^xiv^	68.6 (2)	Cl3D—Cl3B—Na1	94 (2)
O1—Rb3—O4^xv^	68.6 (2)	Na1^xxvi^—Cl3B—Na1	172 (5)
O4^xiv^—Rb3—O4^xv^	70.6 (3)	Cl3A^xxiii^—Cl3B—Cl3D^xxiii^	0.0 (2)
O1—Rb3—Cl2	73.6 (2)	Cl3C—Cl3B—Cl3D^xxiii^	180.000 (11)
O4^xiv^—Rb3—Cl2	126.64 (18)	Cl3D—Cl3B—Cl3D^xxiii^	0.000 (3)
O4^xv^—Rb3—Cl2	126.64 (18)	Na1^xxvi^—Cl3B—Cl3D^xxiii^	94 (2)
O1—Rb3—Na1^xvi^	150.6 (5)	Na1—Cl3B—Cl3D^xxiii^	94 (2)
O4^xiv^—Rb3—Na1^xvi^	138.7 (4)	Cl3B—Cl3C—Cl3A^xxiii^	0.000 (6)
O4^xv^—Rb3—Na1^xvi^	105.8 (6)	Cl3B—Cl3C—Cl3A^xxix^	180.00 (3)
Cl2—Rb3—Na1^xvi^	89.5 (4)	Cl3A^xxiii^—Cl3C—Cl3A^xxix^	180.000 (16)
O1—Rb3—Na1^xvii^	150.6 (5)	Cl3B—Cl3C—Na1^xviii^	124.9 (8)
O4^xiv^—Rb3—Na1^xvii^	105.8 (6)	Cl3A^xxiii^—Cl3C—Na1^xviii^	124.9 (8)
O4^xv^—Rb3—Na1^xvii^	138.7 (4)	Cl3A^xxix^—Cl3C—Na1^xviii^	55.1 (8)
Cl2—Rb3—Na1^xvii^	89.5 (4)	Cl3B—Cl3C—Na1^xxvi^	55.1 (8)
Na1^xvi^—Rb3—Na1^xvii^	48.9 (12)	Cl3A^xxiii^—Cl3C—Na1^xxvi^	55.1 (8)
O1—Rb3—O2^xviii^	45.94 (12)	Cl3A^xxix^—Cl3C—Na1^xxvi^	124.9 (8)
O4^xiv^—Rb3—O2^xviii^	102.02 (18)	Na1^xviii^—Cl3C—Na1^xxvi^	180.0 (6)
O4^xv^—Rb3—O2^xviii^	51.76 (18)	Cl3B—Cl3C—Na1^xxvii^	124.9 (8)
Cl2—Rb3—O2^xviii^	74.91 (16)	Cl3A^xxiii^—Cl3C—Na1^xxvii^	124.9 (8)
Na1^xvi^—Rb3—O2^xviii^	107.0 (6)	Cl3A^xxix^—Cl3C—Na1^xxvii^	55.1 (8)
Na1^xvii^—Rb3—O2^xviii^	152.2 (6)	Na1^xviii^—Cl3C—Na1^xxvii^	110.1 (17)
O1—Rb3—O2	45.94 (12)	Na1^xxvi^—Cl3C—Na1^xxvii^	69.9 (17)
O4^xiv^—Rb3—O2	51.76 (18)	Cl3B—Cl3C—Na1	55.1 (8)
O4^xv^—Rb3—O2	102.03 (18)	Cl3A^xxiii^—Cl3C—Na1	55.1 (8)
Cl2—Rb3—O2	74.91 (16)	Cl3A^xxix^—Cl3C—Na1	124.9 (8)
Na1^xvi^—Rb3—O2	152.1 (6)	Na1^xviii^—Cl3C—Na1	69.9 (17)
Na1^xvii^—Rb3—O2	107.0 (6)	Na1^xxvi^—Cl3C—Na1	110.1 (17)
O2^xviii^—Rb3—O2	91.4 (2)	Na1^xxvii^—Cl3C—Na1	180.0
O1—Rb3—O3^xix^	112.5 (2)	Na1^xxvi^—Cl4—Na1^xxv^	128.1 (15)
O4^xiv^—Rb3—O3^xix^	76.46 (18)	Na1^xxvi^—Cl4—Rb2^xxx^	68.3 (5)
O4^xv^—Rb3—O3^xix^	45.57 (18)	Na1^xxv^—Cl4—Rb2^xxx^	121.6 (6)
Cl2—Rb3—O3^xix^	154.91 (12)	Na1^xxvi^—Cl4—Rb2^xiv^	121.6 (6)
Na1^xvi^—Rb3—O3^xix^	73.8 (4)	Na1^xxv^—Cl4—Rb2^xiv^	68.3 (5)
Na1^xvii^—Rb3—O3^xix^	93.1 (4)	Rb2^xxx^—Cl4—Rb2^xiv^	159.7 (11)
O2^xviii^—Rb3—O3^xix^	91.89 (17)	Na1^xxvi^—Cl4—Rb3^iv^	150.6 (5)
O2—Rb3—O3^xix^	127.56 (17)	Na1^xxv^—Cl4—Rb3^iv^	59.3 (5)
O1—Rb3—O3^ii^	112.5 (2)	Rb2^xxx^—Cl4—Rb3^iv^	83.9 (3)
O4^xiv^—Rb3—O3^ii^	45.57 (18)	Rb2^xiv^—Cl4—Rb3^iv^	87.8 (3)
O4^xv^—Rb3—O3^ii^	76.46 (18)	Na1^xxvi^—Cl4—Rb3^xv^	59.3 (5)
Cl2—Rb3—O3^ii^	154.91 (13)	Na1^xxv^—Cl4—Rb3^xv^	150.6 (5)
Na1^xvi^—Rb3—O3^ii^	93.1 (4)	Rb2^xxx^—Cl4—Rb3^xv^	87.8 (3)
Na1^xvii^—Rb3—O3^ii^	73.8 (4)	Rb2^xiv^—Cl4—Rb3^xv^	83.9 (3)
O2^xviii^—Rb3—O3^ii^	127.56 (17)	Rb3^iv^—Cl4—Rb3^xv^	131.2 (8)
O2—Rb3—O3^ii^	91.89 (17)	Cl3B—Na1—Cl3A^xxiii^	3 (6)
O3^xix^—Rb3—O3^ii^	47.5 (2)	Cl3B—Na1—Cl3C	39 (2)
O1—Rb3—P1^xv^	87.92 (17)	Cl3A^xxiii^—Na1—Cl3C	42 (4)
O4^xiv^—Rb3—P1^xv^	63.49 (14)	Cl3B—Na1—Cl3D	36 (3)
O4^xv^—Rb3—P1^xv^	23.96 (14)	Cl3A^xxiii^—Na1—Cl3D	34 (5)
Cl2—Rb3—P1^xv^	150.05 (11)	Cl3C—Na1—Cl3D	75.5 (15)
Na1^xvi^—Rb3—P1^xv^	96.1 (5)	Cl3B—Na1—Cl4^xxvi^	84 (2)
Na1^xvii^—Rb3—P1^xv^	116.2 (4)	Cl3A^xxiii^—Na1—Cl4^xxvi^	82 (3)
O2^xviii^—Rb3—P1^xv^	75.31 (12)	Cl3C—Na1—Cl4^xxvi^	112.6 (8)
O2—Rb3—P1^xv^	108.96 (12)	Cl3D—Na1—Cl4^xxvi^	58.7 (12)
O3^xix^—Rb3—P1^xv^	24.64 (12)	Cl3B—Na1—Cl4^xxiii^	84 (2)
O3^ii^—Rb3—P1^xv^	54.33 (12)	Cl3A^xxiii^—Na1—Cl4^xxiii^	82 (3)
O1—Rb3—P1^xiv^	87.92 (17)	Cl3C—Na1—Cl4^xxiii^	112.6 (8)
O4^xiv^—Rb3—P1^xiv^	23.96 (14)	Cl3D—Na1—Cl4^xxiii^	58.7 (12)
O4^xv^—Rb3—P1^xiv^	63.49 (14)	Cl4^xxvi^—Na1—Cl4^xxiii^	84.0 (13)
Cl2—Rb3—P1^xiv^	150.05 (11)	Cl3B—Na1—Na1^xviii^	94 (2)
Na1^xvi^—Rb3—P1^xiv^	116.2 (4)	Cl3A^xxiii^—Na1—Na1^xviii^	97 (4)
Na1^xvii^—Rb3—P1^xiv^	96.1 (5)	Cl3C—Na1—Na1^xviii^	55.1 (8)
O2^xviii^—Rb3—P1^xiv^	108.96 (12)	Cl3D—Na1—Na1^xviii^	130.5 (16)
O2—Rb3—P1^xiv^	75.31 (12)	Cl4^xxvi^—Na1—Na1^xviii^	137.9 (6)
O3^xix^—Rb3—P1^xiv^	54.33 (12)	Cl4^xxiii^—Na1—Na1^xviii^	137.9 (6)
O3^ii^—Rb3—P1^xiv^	24.64 (12)	Cl3B—Na1—Cl3D^xxiii^	46 (3)
P1^xv^—Rb3—P1^xiv^	47.69 (8)	Cl3A^xxiii^—Na1—Cl3D^xxiii^	43 (4)
O4^ii^—Cu1—O4^xx^	171.8 (5)	Cl3C—Na1—Cl3D^xxiii^	85.3 (12)
O4^ii^—Cu1—O2^xiv^	92.2 (3)	Cl3D—Na1—Cl3D^xxiii^	10 (2)
O4^xx^—Cu1—O2^xiv^	90.0 (3)	Cl4^xxvi^—Na1—Cl3D^xxiii^	52.9 (10)
O4^ii^—Cu1—O2^iv^	90.0 (3)	Cl4^xxiii^—Na1—Cl3D^xxiii^	52.9 (10)
O4^xx^—Cu1—O2^iv^	92.2 (3)	Na1^xviii^—Na1—Cl3D^xxiii^	140.4 (11)
O2^xiv^—Cu1—O2^iv^	148.5 (4)	Cl3B—Na1—Rb3^xxxi^	110.0 (11)
O4^ii^—Cu1—Cl1	85.9 (2)	Cl3A^xxiii^—Na1—Rb3^xxxi^	111.1 (18)
O4^xx^—Cu1—Cl1	85.9 (2)	Cl3C—Na1—Rb3^xxxi^	91.1 (8)
O2^xiv^—Cu1—Cl1	105.8 (2)	Cl3D—Na1—Rb3^xxxi^	120.4 (5)
O2^iv^—Cu1—Cl1	105.8 (2)	Cl4^xxvi^—Na1—Rb3^xxxi^	153.5 (11)
O4^ii^—Cu1—Rb3^xv^	141.1 (2)	Cl4^xxiii^—Na1—Rb3^xxxi^	75.6 (5)
O4^xx^—Cu1—Rb3^xv^	45.3 (2)	Na1^xviii^—Na1—Rb3^xxxi^	65.5 (6)
O2^xiv^—Cu1—Rb3^xv^	56.4 (2)	Cl3D^xxiii^—Na1—Rb3^xxxi^	121.2 (5)
O2^iv^—Cu1—Rb3^xv^	105.5 (2)	Cl3B—Na1—Rb3^xxxii^	110.0 (11)
Cl1—Cu1—Rb3^xv^	121.90 (3)	Cl3A^xxiii^—Na1—Rb3^xxxii^	111.1 (18)
O4^ii^—Cu1—Rb3^iv^	45.3 (2)	Cl3C—Na1—Rb3^xxxii^	91.1 (8)
O4^xx^—Cu1—Rb3^iv^	141.1 (2)	Cl3D—Na1—Rb3^xxxii^	120.4 (5)
O2^xiv^—Cu1—Rb3^iv^	105.5 (2)	Cl4^xxvi^—Na1—Rb3^xxxii^	75.6 (5)
O2^iv^—Cu1—Rb3^iv^	56.4 (2)	Cl4^xxiii^—Na1—Rb3^xxxii^	153.5 (11)
Cl1—Cu1—Rb3^iv^	121.90 (3)	Na1^xviii^—Na1—Rb3^xxxii^	65.5 (6)
Rb3^xv^—Cu1—Rb3^iv^	116.19 (7)	Cl3D^xxiii^—Na1—Rb3^xxxii^	121.2 (5)
O3—Cu2—O3^ix^	87.1 (4)	Rb3^xxxi^—Na1—Rb3^xxxii^	117.5 (10)
O3—Cu2—O3^xxi^	169.6 (5)	Cl3B—Na1—Rb2^xiii^	134 (3)
O3^ix^—Cu2—O3^xxi^	92.0 (4)	Cl3A^xxiii^—Na1—Rb2^xiii^	131 (5)
O3—Cu2—O3^xxii^	92.0 (4)	Cl3C—Na1—Rb2^xiii^	172.8 (14)
O3^ix^—Cu2—O3^xxii^	169.6 (5)	Cl3D—Na1—Rb2^xiii^	97.3 (18)
O3^xxi^—Cu2—O3^xxii^	87.1 (4)	Cl4^xxvi^—Na1—Rb2^xiii^	62.6 (7)
O3—Cu2—Cl2^xii^	95.2 (2)	Cl4^xxiii^—Na1—Rb2^xiii^	62.6 (7)
O3^ix^—Cu2—Cl2^xii^	95.2 (2)	Na1^xviii^—Na1—Rb2^xiii^	132.1 (6)
O3^xxi^—Cu2—Cl2^xii^	95.2 (2)	Cl3D^xxiii^—Na1—Rb2^xiii^	87.5 (14)
O3^xxii^—Cu2—Cl2^xii^	95.2 (2)	Rb3^xxxi^—Na1—Rb2^xiii^	92.7 (5)
O2—P1—O4	115.7 (4)	Rb3^xxxii^—Na1—Rb2^xiii^	92.7 (5)
O2—P1—O3^vi^	111.7 (4)	Cl3B—Na1—Na1^xxvi^	4 (2)
O4—P1—O3^vi^	112.6 (4)	Cl3A^xxiii^—Na1—Na1^xxvi^	7 (4)
O2—P1—O1	104.4 (4)	Cl3C—Na1—Na1^xxvi^	34.9 (8)
O4—P1—O1	104.4 (4)	Cl3D—Na1—Na1^xxvi^	40.5 (16)
O3^vi^—P1—O1	107.0 (4)	Cl4^xxvi^—Na1—Na1^xxvi^	87.1 (7)
O2—P1—Rb3^x^	165.7 (3)	Cl4^xxiii^—Na1—Na1^xxvi^	87.1 (7)
O4—P1—Rb3^x^	52.2 (3)	Na1^xviii^—Na1—Na1^xxvi^	89.998 (3)
O3^vi^—P1—Rb3^x^	71.6 (3)	Cl3D^xxiii^—Na1—Na1^xxvi^	50.4 (11)
O1—P1—Rb3^x^	87.3 (3)	Rb3^xxxi^—Na1—Na1^xxvi^	108.2 (4)
O2—P1—Rb2	52.0 (3)	Rb3^xxxii^—Na1—Na1^xxvi^	108.2 (4)
O4—P1—Rb2	142.1 (3)	Rb2^xiii^—Na1—Na1^xxvi^	137.9 (6)

Symmetry codes: (i) *x*, −*y*, *z*−1/2; (ii) −*x*, *y*, −*z*+1/2; (iii) −*y*, −*x*, *z*−1/2; (iv) *y*, *x*, −*z*+1/2; (v) *y*, *x*, *z*−1/2; (vi) −*y*, −*x*, −*z*+1/2; (vii) −*x*, *y*, *z*−1/2; (viii) *x*, −*y*, −*z*+1/2; (ix) *y*−1/2, *x*+1/2, *z*; (x) −*y*, *x*, *z*; (xi) *x*−1/2, *y*+1/2, −*z*+1/2; (xii) −*x*−1/2, −*y*+1/2, −*z*+1/2; (xiii) −*x*+1/2, −*y*+1/2, −*z*+1/2; (xiv) *y*, −*x*, *z*; (xv) *y*, −*x*, −*z*+1; (xvi) *y*, −*x*+1, −*z*+1; (xvii) *y*, −*x*+1, *z*; (xviii) *x*, *y*, −*z*+1; (xix) −*x*, *y*, *z*+1/2; (xx) −*x*, −*y*, *z*; (xxi) *y*−1/2, *x*+1/2, −*z*; (xxii) *x*, *y*, −*z*; (xxiii) *y*+1/2, −*x*+1/2, −*z*+1/2; (xxiv) −*y*+1/2, *x*−1/2, *z*−1/2; (xxv) −*y*+1/2, *x*−1/2, −*z*+1/2; (xxvi) −*x*+1, −*y*, *z*; (xxvii) −*x*+1, −*y*, −*z*+1; (xxviii) *y*−1/2, *x*+1/2, −*z*+1; (xxix) −*y*+1/2, *x*−1/2, *z*+1/2; (xxx) *x*+1/2, *y*−1/2, −*z*+1/2; (xxxi) *x*+1/2, −*y*+1/2, *z*; (xxxii) −*y*+1, *x*, *z*.
